# Atrial Fibrillation Burden and Clinical Outcomes After Catheter Ablation vs Lifestyle Modification With Antiarrhythmic Drugs

**DOI:** 10.1016/j.jacadv.2026.103109

**Published:** 2026-08-06

**Authors:** Zuzana Hejdukova, Dalibor Herman, Marek Hozman, Tomas Roubicek, Ivan Ranic, Stepan Havranek, Milan Dusik, Kristyna Souza–Lopes, Jan Chovancik, Jana Vesela, Veronika Bulkova, Klara Benesova, Pavel Osmancik

**Affiliations:** a Cardiocenter, Third Faculty of Medicine, Charles University, Prague, and University Hospital Kralovske Vinohrady, Prague, Czech Republic; b Department of Cardiology, Regional Hospital Liberec, Czech Republic; c Department of Cardiology, Cardiocenter, Hospital Podlesí a.s., Trinec, Czech Republic; d Cardiocenter, Second Internal Clinic–Cardiology and Angiology, Charles University, General Faculty Hospital, Prague, Czech Republic; e Department of Cardiology, Neuron Medical Center, Hospital Brno, Czech Republic; f Masaryk University, Institute of Biostatistics and Analyses, Czech Republic

**Keywords:** atrial fibrillation, catheter ablation, lifestyle modification, weight loss

## Abstract

**Background:**

PRAGUE–25 compared catheter ablation (CA) with lifestyle modification combined with antiarrhythmic drugs (LFM + AADs) in obese patients with atrial fibrillation (AF).

**Objectives:**

The objective of the study was to evaluate: 1) AF burden; and 2) clinical outcomes in CA vs LFM + AAD per-protocol populations enrolled in the PRAGUE–25 trial.

**Methods:**

This per-protocol analysis included patients who adhered to the assigned treatment strategy (patients’ crossing over between treatment strategies were excluded at the time of crossover). AF burden was assessed by 7-day Holter monitoring at baseline and 6, 9, and 12 months after randomization. Clinical outcomes included AF-related hospitalizations or emergency visits and major adverse cardiovascular events (composite of stroke, cardiovascular death, or heart failure hospitalization) during the study course.

**Results:**

Of the 203 randomized patients, 96 CA and 83 LFM + AAD patients were included in the per-protocol analysis. The mean age was 60 ± 9 years, 68.5% were men, and the mean body mass index of 35 ± 3 kg/m^2^. Baseline AF burden was similar between groups (median [IQR] 3.2% [0.0-98.0] vs 5.8% [0.0-100.0]; *P* = 0.39). AF burden was lower in the CA group at 6, 9, and 12 months (all *P* < 0.001). AF-related hospitalizations or emergency visits occurred in 7.0% vs 15.5% (*P* = 0.026), whereas major adverse cardiovascular events were rare and similar between groups (1.0% vs 1.9%; *P* = 0.99).

**Conclusions:**

In this per-protocol analysis, CA resulted in superior rhythm control compared with LFM + AAD therapy. AF burden reduction was accompanied by fewer AF-related hospitalizations or emergency visits. (PRAGUE-25 Trial: Catheter Ablation vs. AADs and Risk Factor Modification; NCT04011800).

Obesity substantially increases the risk of atrial fibrillation (AF). Large population analyses demonstrate a clear dose-response relationship, with each 5-kg/m^2^ increase in body mass index (BMI) associated with a 20% to 30% higher incidence of AF.^1^

Lifestyle Modification (LFM), including weight reduction, increased physical activity, reduced alcohol intake, and additional cardiometabolic risk optimizations, can significantly affect AF treatment. Several observational studies have shown that LFM measures substantially improve AF freedom in patients with or without ablation. However, most of these studies were nonrandomized, lacked a control group, and tended to include highly motivated AF patients.^2^^,^^3^

Recently, the randomized PRAGUE–25 trial demonstrated the superiority of catheter ablation (CA) compared with treatment based on a combination of LFM and antiarrhythmic drugs (AADs) for sinus rhythm maintenance. Patients were monitored using 7-day Holter recordings at baseline and every 3 months during the first year. The primary outcome was AF recurrence, defined as any episode of AF or atrial tachycardia lasting more than 30 seconds. The analysis was conducted according to the intention-to-treat (ITT) principle.^4^

However, as demonstrated in recent studies, AF burden is a clinically more important parameter than assessment of the first isolated AF episode lasting longer than 30 seconds. In the original PRAGUE–25 publication, only AF burden at 12 months (as one of the secondary endpoints) was reported, and the analysis was conducted according to the ITT principle. Unlike a time-to-event analysis, in which patients are censored at AF recurrence, AF burden assessment at 12 months could be strongly affected by previous crossover in the ITT analysis. Therefore, to assess the true effect of CA and LFM + AAD, a per-protocol analysis is more appropriate.

The aim of the present subanalysis of the PRAGUE–25 study is to evaluate AF burden in the per-protocol population at each milestone at which Holter monitoring was performed (6, 9, and 12 months during the first year). In addition to AF burden, another objective of this report is to present the clinical outcomes of the study patients. These outcomes were prespecified as secondary endpoints but were not published in the primary report. The study design and main findings are summarized in the Central Illustration.

**Central Illustration:**
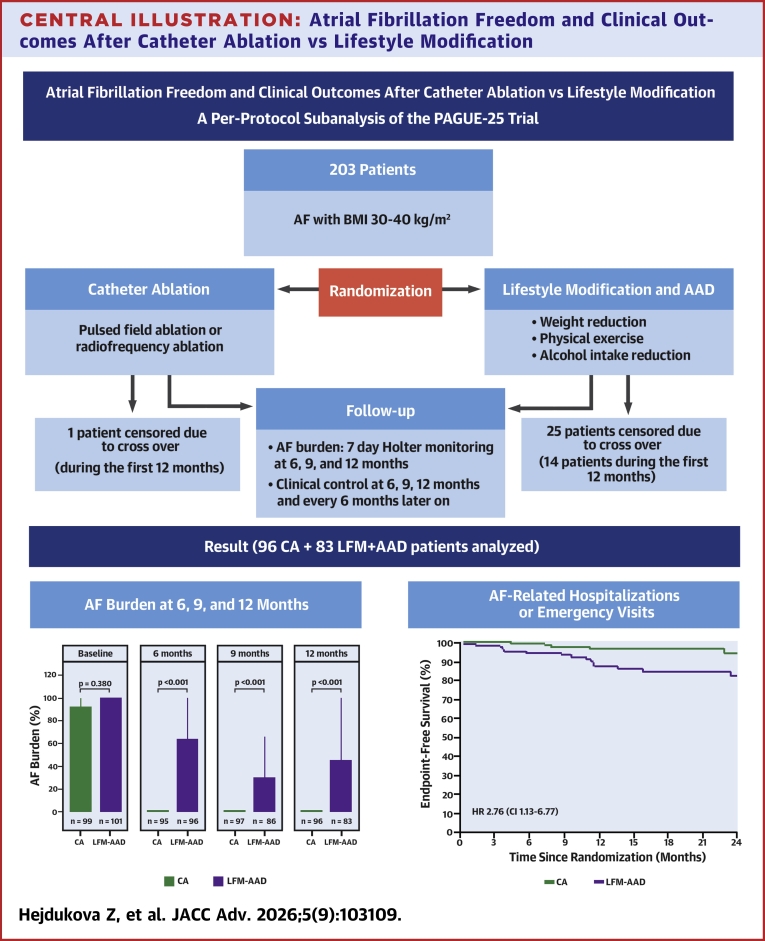
**Atrial Fibrillation Freedom and Clinical Outcomes After Catheter Ablation vs Lifestyle Modification** Overview of the PRAGUE-25 subanalysis in obese patients with atrial fibrillation randomized to catheter ablation or lifestyle modification combined with antiarrhythmic drug therapy. Catheter ablation was associated with greater reduction in atrial fibrillation burden and favorable clinical outcomes during follow-up. AAD = antiarrhythmic drug; AF = atrial fibrillation, BMI = body mass index; CA = catheter ablation; LFM = lifestyle modification.

## Methods

### Study design

This analysis reports: 1) the 12-month postrandomization AF burden; and 2) AF-related outcomes for the entire study population in the per-protocol cohort of the PRAGUE–25 study. PRAGUE–25 was a multicenter, randomized, investigator-initiated noninferiority trial that compared CA with a treatment strategy combining structured LFM and AAD therapy in patients with AF and a BMI between 30 to 40 kg/m^2^. The study was conducted at five sites in the Czech Republic and received ethical approval from the University Hospital Kralovske Vinohrady Ethics Committee and from the ethics committees of all participating centers. Written informed consent was obtained from all participants before enrollment. Comprehensive descriptions of the study design and the primary outcomes have been published previously.^4^^,^^5^

### Original and present patient population

The PRAGUE–25 trial enrolled patients with symptomatic AF (paroxysmal, persistent, or long-standing persistent) and a BMI between 30 and 40 kg/m^2^, all of whom provided written informed consent. Major exclusion criteria included a history of tachycardia-induced cardiomyopathy, BMI >40 kg/m^2^, left ventricular ejection fraction ≤40%, untreated coronary artery disease, left atrial diameter >60 mm, an indication for bariatric surgery, age ≥75 years, contraindications to AADs, or significant limitations in physical activity. A complete list of eligibility criteria is provided in the original publication.^4^

Eligible participants were randomly assigned in a 1:1 ratio to CA or LFM combined with AAD therapy (LFM + AAD). Randomization was implemented through a secure web-based system using permuted blocks and was stratified according to BMI category, sex, and AF type.

This subanalysis was conducted in the per-protocol population of the PRAGUE–25 trial and examined both AF burden and clinical outcomes. To ensure that rhythm outcomes reflected the originally assigned treatment strategy, patients who crossed over between study groups were censored at the time of crossover. AF burden was assessed using 7-day Holter monitoring performed at 6, 9, and 12 months after randomization (ie, during the first 12 months of follow-up). Patients with insufficient rhythm monitoring (fewer than 5 days of analyzable data per protocol) were also excluded.

### Numbers of patients analyzed at individual time points for AF burden analysis

Patients contributed AF-burden data only for the period during which they remained adherent to their originally assigned treatment strategy and were censored at the time of crossover. Because changes in AF burden over time were also assessed on an individual patient level, baseline Holter monitoring was mandatory; therefore, patients without baseline Holter data had to be excluded from the AF burden analysis.

Of the 203 randomized patients, 200 entered in the AF-burden analysis (99 patients in the CA and, and 101 patients in the LFM + AAD group), as 3 patients did not undergo baseline Holter monitoring.

With regard to crossover and analysis of the LFM + AAD group, 2 patients crossed over before 6 months, 8 patients between 6 and 9 months, and 4 patients between 9 and 12 months. Consequently, 99, 91, and 87 patients remained in follow-up at 6, 9, and 12 months, respectively. Because Holter monitoring data were missing in 3, 5, and 4 patients at these respective milestones, 96, 86, and 83 patients were ultimately included in the AF burden analysis at 6, 9, and 12 months.

With regard to crossover in the CA group, only 1 patient crossed over during the course of the study (before 6 months). Consequently, 98 patients remained in follow-up at each milestone (6, 9, and 12 months). Because Holter monitoring data were missing in 3, 1, and 2 patients at these respective time points, 95, 97, and 96 patients were ultimately included in the AF burden analysis at 6, 9, and 12 months, respectively.

**Figure 1:**
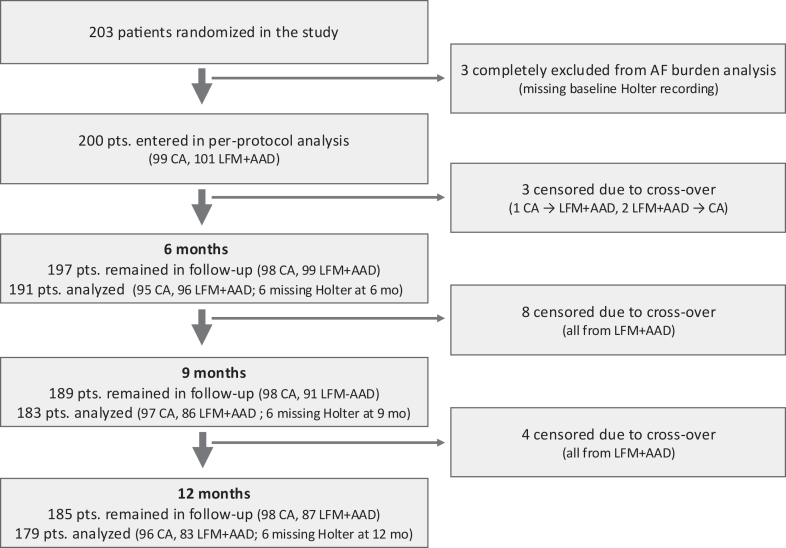
**CONSORT Flow Diagram of the Per-Protocol Population for the AF-Burden Analysis** The diagram shows the number of patients included at baseline and analyzed at each follow-up milestone at 6, 9, and 12 months. Patients who crossed over between treatment strategies were censored at the time of crossover, and patients with missing Holter data were excluded from the respective time-point analysis. AAD = antiarrhythmic drug; AF = atrial fibrillation; CA = catheter ablation; LFM = lifestyle modification.

The CONSORT diagram (Figure 1) provides a detailed overview of patient flow throughout the study, including the number of patients analyzed at each follow-up milestone.

### Numbers of patients analyzed at individual time points for clinical outcomes

Clinical outcomes were defined as AF-related outcomes and major adverse cardiovascular (CV) events (MACE). AF-related outcomes included AF-related hospitalizations or AF-related emergency department visits; MACE was defined as a composite of stroke, CV death, and heart failure (HF) hospitalization.

For the analysis of clinical outcomes, all 203 randomized patients were included according to their originally allocated treatment groups (100 in the CA group and 103 in the LFM + AAD group). Patients were followed from the time of randomization until the occurrence of the respective clinical endpoint or censoring. In contrast to the AF-burden analysis, no blanking period was applied for clinical outcomes. Likewise, unlike in the AF burden analysis, the absence of Holter monitoring was not a reason for exclusion from the clinical outcomes analysis. In accordance with the per-protocol approach, patients who crossed over between treatment strategies were censored at the time of crossover, and only events occurring before crossover were included in the analysis.

Finally, also in contrast to the AF burden analysis, clinical outcomes were assessed throughout the entire follow-up period rather than being limited to the first 12 months. Accordingly, the cohorts for AF-burden and clinical outcome analyses slightly differed in terms of the number of analyzed patients. With regard to follow-up duration, the protocol-specified minimum follow-up period was 12 months with longer follow-up available for those enrolled earlier in the study.

### Study procedures

#### Catheter ablation group

Participants randomized to the CA group underwent CA within 6 weeks of enrollment. A 3-month postprocedure interval was defined as the blanking period. All AADs had to be discontinued by the end of the blanking period. Restarting AAD therapy after the blanking period was permitted only in the presence of recurrent arrhythmia and was classified as treatment failure.

#### LFM + AAD group

The LFM + AAD strategy was initiated within 4 weeks of randomization. LFM was delivered under the guidance of a multidisciplinary team consisting of dietitians and physiotherapists, with the full structure of the program described previously.^5^^,^^6^ Patients attended scheduled in-person consultations and received additional follow-up via telephone, complemented by digital support through the OBEFIS mobile application.

The lifestyle program included individualized dietary counseling, recommendations to reduce alcohol consumption, and personalized exercise plans prepared by physiotherapists. AAD therapy was also introduced within 4 weeks after randomization, with dose adjustments performed during the first 3 months. Unlike in the CA group, AAD therapy could be continued throughout the entire follow-up period. As in the CA group, the initial 3 months after starting the LFM program were defined as the blanking period.

#### Baseline and follow-up examinations

A *baseline* 7-day Holter electrocardiogram was obtained within 4 weeks after randomization together with the initial study evaluations, which included transthoracic echocardiography and assessment of quality of life using the Atrial Fibrillation Effect on QualiTy-of-life questionnaire, as detailed in the original report.^5^ Follow-up visits were scheduled at 3, 6, 9, and 12 months after either the CA procedure or the initiation of the full LFM + AAD program. At the 3-month visit, all patients who remained in AF, regardless of treatment assignment, underwent electrical cardioversion; the first 3 months after randomization were defined as a blanking period in both groups, during which AF recurrences were not considered as study endpoints. This approach was intended to ensure sinus rhythm at the start of the subsequent follow-up period, whereas during further follow-up cardioversion was performed only when clinically indicated and was not scheduled in relation to Holter monitoring. Seven-day Holter monitoring was subsequently repeated at 3-month intervals throughout the first year following randomization and every 6 months thereafter. At least 12 months of follow-up after randomization was required for all patients according to the study protocol.

#### Endpoint definition

AF burden was defined as the percentage of time patients spent in AF during each 7-day Holter monitoring (baseline, at 6, 9, and 12 months). The delta AF burden (ΔAF burden) was defined as the difference between AF burden at each follow-up milestone (6, 9, and 12 months) and the baseline AF burden value. For the purposes of this analysis, ΔAF burden was first calculated for each individual patient at each predefined milestone, and the resulting ΔAF burden values were subsequently compared between the 2 groups. Finally, relative reduction of AF burden was defined as: (1 − [AF burden at the particular milestone/AF burden at baseline]) × 100. All AF burdens (absolute, relative, and Δ) were calculated at 6, 9, and 12 months; patients who crossed over were censored at the time of crossover.

Unlike AF-burden analysis, the clinical outcomes were assessed over the entire study period for each patient until the end of the study. As in the AF burden analysis, patients who crossed over were censored at the time of crossover. AF-related clinical outcomes were defined as hospitalizations or emergency department visits for AF. MACE were defined as a composite of stroke, CV death, or HF hospitalization.

#### Statistical analysis

Normality of data distribution was assessed using the Shapiro-Wilk test. Baseline continuous characteristics were compared using the Mann-Whitney *U* test, whereas categorical variables were compared using the chi-square test or Fisher exact test. Variables with a normal distribution are presented as mean ± SD, whereas non-Gaussian variables are reported as median and IQR. Because AF burden data were non-normally distributed, they were analyzed using beta regression. Within-group comparison of AF burden was initially performed using the Friedman test, followed by post hoc Wilcoxon signed rank tests.

Subsequently, AF burden at baseline was compared with AF burden at 6, 9, and 12 months within each group, with results adjusted for multiplicity using the Holm correction. Between-group comparisons of AF burden for the CA and LFM + AAD groups at each analyzed milestone (ie, 6, 9, and 12 months) were performed using the Mann-Whitney *U* test, with Holm corrections applied for multiplicity.

Kaplan-Meier estimates were generated to visualize clinical outcomes during the 24-month follow-up. A Cox proportional hazards regression model was used to evaluate the association between treatment strategy and the risk of the studied endpoint, with results expressed as HRs and 95% CIs. A 2-sided *P* value <0.05 was considered statistically significant. Statistical analyses were performed using R (version 4.3.2; R Core Team, R Foundation for Statistical Computing). As a sensitivity analysis, between-group comparisons of 12-month AF burden, VO_2_ max, and quality-of-life outcomes were performed in the per-protocol populations with adjustment for the corresponding baseline values; details are provided in the Supplemental Appendix.

## Results

### Patients

Between May 2021 and November 2023, a total of 203 patients with AF were enrolled in the PRAGUE–25 trial. For AF-burden analysis, 200 patients with available baseline Holter monitoring were included (99 in the CA group and 101 in the LFM + AAD group). During the first 12 months, 15 patients crossed over between treatment strategies (1 from the CA group to the LFM + AAD group and 14 from the LFM + AAD group to the CA group) and were censored at the time of crossover. The number of patients analyzed at individual time points is provided in detail in the Figure 1 and in the Methods section.

For the analysis of clinical outcomes, all 203 patients (100 in the CA group and 103 in the LFM + AAD group) were included according to their originally allocated treatment groups. Over the entire follow-up period, 26 patients changed treatment strategies (1 from CA to LFM + AAD and 25 from LFM + AAD to CA). The median follow-up for clinical assessment was 22.8 months (IQR: 14.7-30.6).

At baseline, median body weight was comparable between groups: 110 kg (99.8-120.0) in the CA group and 107 kg (95.0-122.0) in the LFM + AAD group. From baseline to 12 months, median body weight remained stable in the CA group (+0.2 kg, IQR: −3.3 to 2.7) but decreased significantly in the LFM + AAD group (−6.5 kg, IQR: −10.8 to −1.3; *P* < 0.001).

The median follow-up was 21.0 months (14.9-31.3) in the CA group and 19.1 months (12.5-27.3) in the LFM + AAD group. Baseline characteristics and characteristics of patients included in the 12-month AF-burden analysis are shown in Table 1.

**Table 1:** Baseline Characteristics of Analyzed Patients

	CA Group (n = 99)	LFM + AAD Group (n = 101)	*P* Value
Age, y	60 ± 9	59.8 ± 9	0.995
Sex			0.924
Male	67 (67.7)	70 (69.3)	
Body weight, kg	110 ± 15	109 ± 17	0.578
Body mass index, kg/m^2^	35 ± 2.9	34.8 ± 3.2	0.697
AF type			0.999
Paroxysmal	55 (55.6)	56 (55.4)	
Persistent	39 (39.4)	40 (39.6)	
Long-standing persistent	5 (5.1)	5 (5.0)	
Medical history			
Heart failure	13 (13.1)	10 (9.9)	0.621
Hypertension	84 (84.8)	85 (84.2)	1.000
Diabetes mellitus	19 (19.2)	29 (28.7)	0.158
CHA_2_DS_2_-VASc score			0.801
Mean	2.0 ± 1.2	2.0 ± 1.2	
0	7 (7.1)	9 (8.9)	
1	32 (32.3)	28 (27.7)	
2	27 (27.3)	30 (29.7)	
3	20 (20.2)	20 (19.8)	
>3	12 (12.1)	14 (13.9)	
History of electrical cardioversion	36 (36.4)	46 (45.5)	0.240
Baseline medication			
AADs	58 (58.6)	68 (67.3)	0.257
Oral anticoagulants	89 (89.9)	90 (89.1)	1.000
BBs	75 (75.8)	84 (83.2)	0.262
Echocardiography			
Left atrium diameter, mm	45 ± 6.1	45.6 ± 5.7	0.405
LVEF, %	59 ± 8.2	61.0 ± 5.6	0.035

Values are n (%) or mean ± SD. *P* values were calculated with the use of Fisher test or Mann-Whitney U-test. CHA_2_DS_2_-VASc and AF type Chi-squared test.

AF = atrial fibrillation; AAD = antiarrhythmic drug; BB = beta-blocker; CA = catheter ablation; LFM = lifestyle modification; LVEF = left ventricular ejection fraction.

### AF burden: within-group and between-groups comparisons

**Figure 2:**
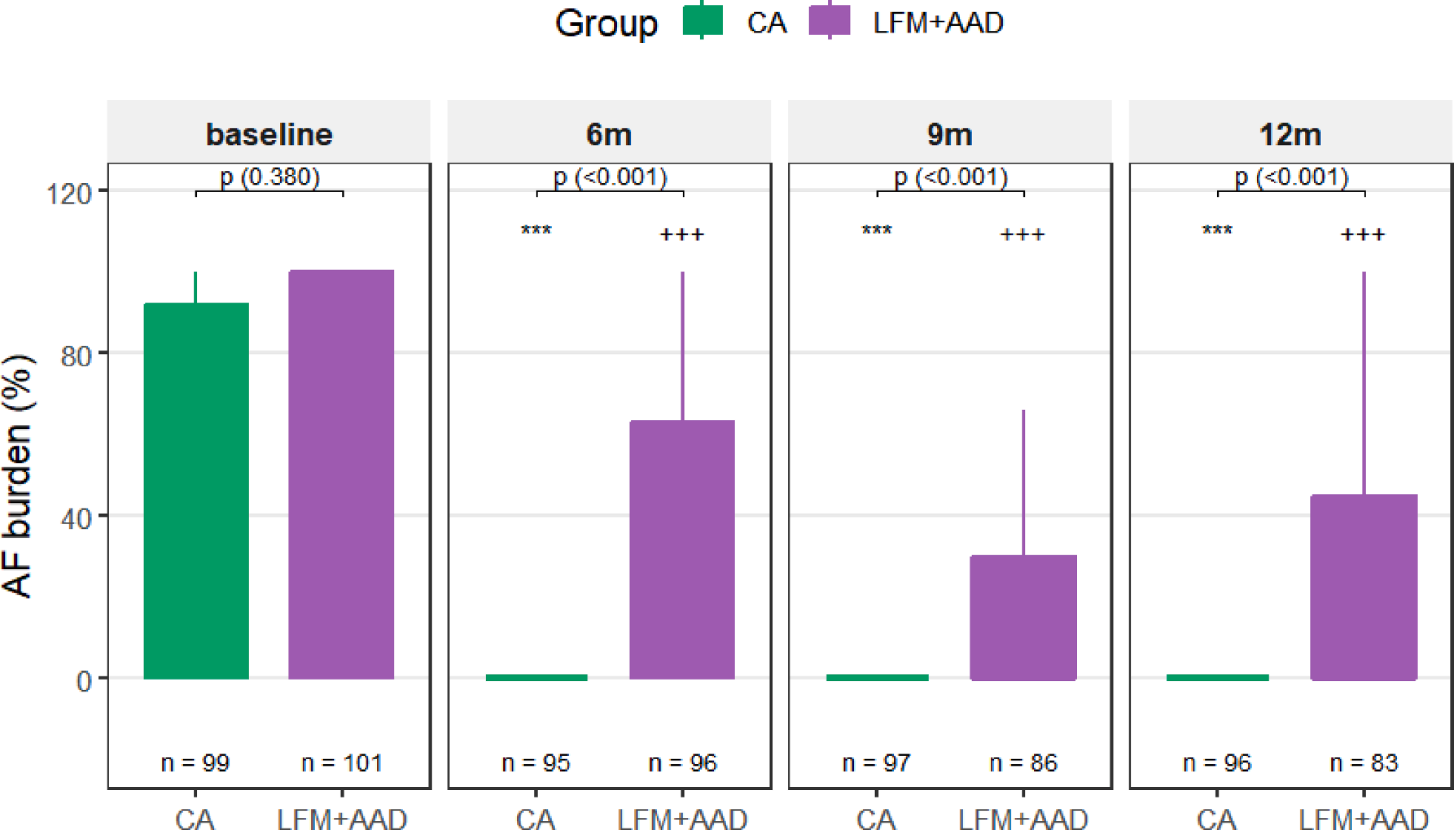
**AF Burden by Treatment Group** AF burden (%) at baseline and at 6, 9, and 12 months is shown for patients treated with CA or LFM + AAD. Data are present as box-and-whisker plots with between-group *P* values displayed for each time point. Boxes indicate medians and IQRs; whiskers extend to the most extreme values within 1.5 × the IQR. Within-group comparisons (AF burden at each milestone vs baseline) are displayed with ∗ for the CA group (∗∗∗*P* < 0.001), and with + for the LFM + AAD group (+++ *P* < 0.001). Abbreviations as in Figure 1.

At baseline, AF burden did not differ significantly between the 2 treatment groups (Figure 2). During follow-up, AF burden decreased significantly in both groups; however, the magnitude and temporal pattern of reduction differed substantially between the 2 groups.

Overall changes in AF burden across time points were significant in both groups (Friedman test: CA, chi-square(3) = 67.8, *P* < 0.001; LFM + AAD, chi-square(3) = 16.6, *P* < 0.001). With regard to the within-group comparison in the CA group, AF burden decreased from 3.2 (IQR: 0-92) at baseline to 0.0 (IQR: 0-0) at 6 months (*P* < 0.001), 0.0 (IQR: 0-0) at 9 months (*P* < 0.001), and to 0.0 (IQR: 0-0) at 12 months (*P* < 0.001). In the LFM + AAD group, AF burden decreased from 5.78 (IQR: 0-100) at baseline to 1.79 (IQR: 0-63.2) at 6 months (*P* = 0.081), 0.0 (IQR: 0-30.0) at 9 months (*P* = 0.002), and to 0.0 (IQR: 0-44.8) at 12 months (*P* = 0.032).

Importantly, with regard to the between-group comparison, AF burden was significantly lower with CA than with LFM + AAD at each analyzed milestone (all *P* < 0.001 for comparisons at 6, 9, and 12 months) (Figure 2, Table 2).

**Table 2:** AF Burden and Delta AF Burden

	Baseline AF Burden		6 Mo AF Burden		6 Mo ΔAF Burden		9 Mo AF Burden		9 Mo ΔAF Burden		12 Mo AF Burden		12 Mo ΔAF Burden	
	Mean ± SD	Median (IQR)	Mean ± SD	Median (IQR)	Mean ± SD	Median (IQR)	Mean ± SD	Median (IQR)	Mean ± SD	Median (IQR)	Mean ± SD	Median (IQR)	Mean ± SD	Median (IQR)
CA	31.1 ± 42.6	3.2 (0.0, 98.0)	9.1 ± 25.8	0.0 (0.0, 0.0)	−22.4 ± 41.7	0.0 (−28.4, 0.0)	10.2 ± 28.8	0.0 (0.0, 0.0)	−21.4 ± 46.2	0.0 (−42.9, 0)	11.9 ± 31.2	0.0 (0.0, 0.0)	−19.9 ± 47.4	0.0 (−36.6, 0.0)
LFM + AAD	35.9 ± 44.1	5.8 (0.0, 100.0)	29.7 ± 41.4	1.79 (0.0, 69.95)	−5.4 ± 37.8	0.0 (−7.7, 0.0)	24.3 ± 39.7	0 (0.0, 33.7)	−8.9 ± 39.3	0.0 (−10.8, 0)	24.4 ± 38.6	0.0 (0.0, 45.5)	−8.9 ± 40.1	0.0 (−11.9, 0.0)
*P* value		0.39		<0.001		0.003		<0.001		0.064		<0.001		0.017

Values are based on patients with available data at each time point: baseline (CA, n = 99; LFM + AAD, n = 101), 6 months (CA, n = 95; LFM + AAD, n = 96), 9 months (CA, n = 97; LFM + AAD, n = 86), and 12 months (CA, n = 96; LFM + AAD, n = 83).

ΔAF burden = AF burden at particular time – AF burden at baseline; other abbreviations as in Table 1.

When expressed as a relative reduction, the median relative reduction in AF burden compared with baseline was 100% (IQR: 86.5-100) at 6 months, 100% (IQR: 94.2-100)at 9 months, and 100% (IQR: 94.3-100) at 12 months in the CA group, and 20.1% (IQR: 0-100), 65.1% (IQR: 0-100), and 59.8% (IQR: 0-100), at 6, 9, and 12 months, respectively, in the LFM + AAD group. Consistent with absolute AF-burden results, the relative reduction of AF burden was also significantly greater with CA at each analyzed milestone (*P* < 0.001 for all comparisons at 6, 9, and 12 months). An adjusted sensitivity analysis of AF burden at 12 months accounting for baseline AF burden and baseline AF type (paroxysmal vs nonparoxysmal) confirmed the robustness of these findings (Supplemental Table 1). Transitions in AF-burden patterns (ie the change from paroxysmal to nonparoxysmal AF type) between baseline and follow-up was not different between the groups; results are provided in the Supplemental Material (Supplemental Table 2).

### Delta AF burden (ΔAF burden)

**Figure 3:**
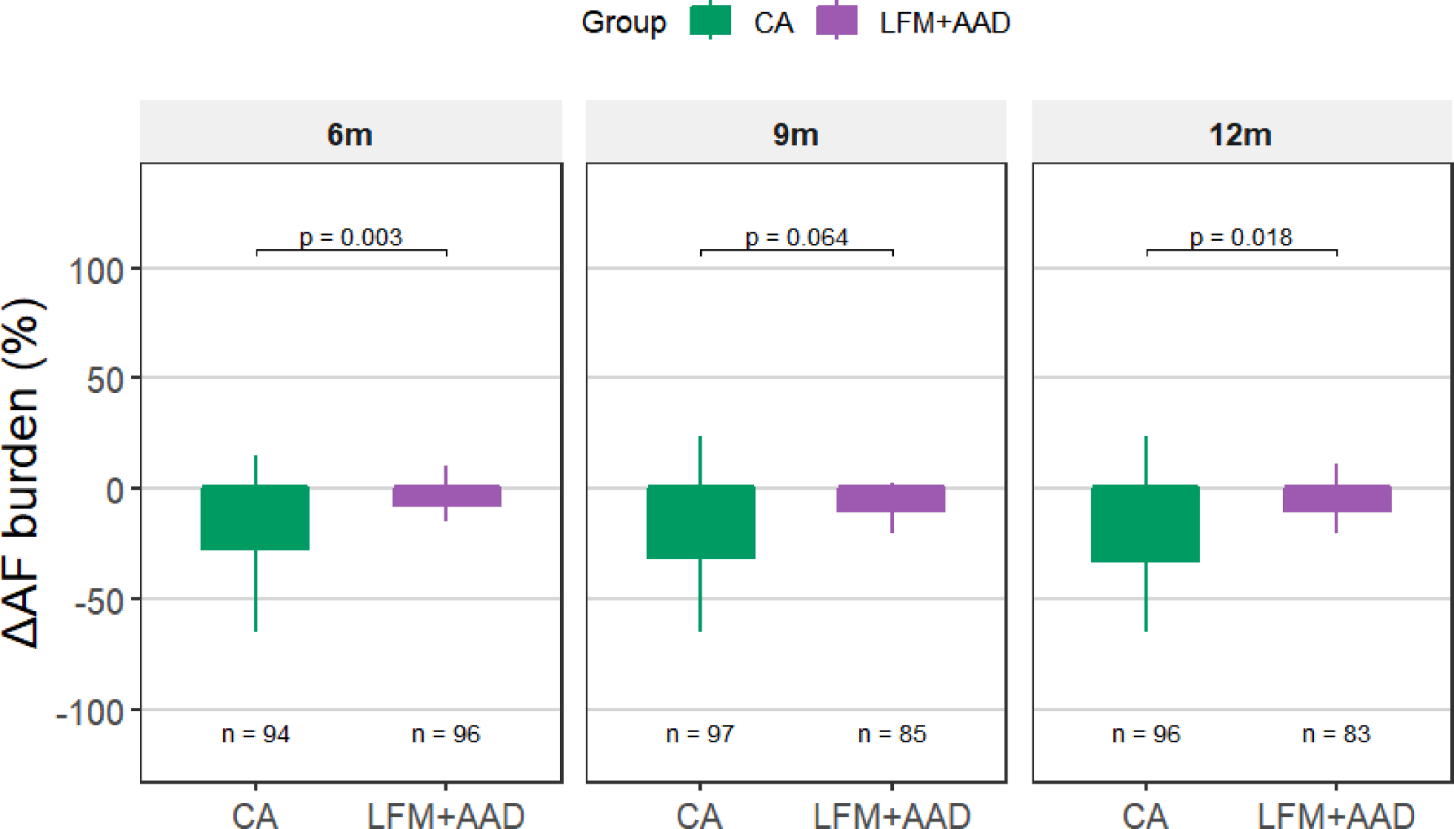
**ΔAF Burden by Treatment Group** The difference between AF burden at each follow-up milestone and baseline (ΔAF burden, %) at 6, 9, and 12 months is shown for patients treated with CA or LFM + AAD. Data are presented as box-and-whisker plots with between-group *P* values displayed for each time point. Boxes indicate medians and IQRs; whiskers extend to the most extreme values within 1.5 × the IQR. Abbreviations as in Figure 1.

As noted, ΔAF burden was defined as the difference between AF burden at each milestone and AF burden at baseline. The results of ΔAF burden are shown in Table 2 and Figure 3. ΔAF burden was 0.0 (−28.4, 0.0) in the CA group vs 0.0 (−7.7, 0.0) in the LFM + AAD group at 6 months (*P* = 0.003 for the between-group comparison), 0.0 (−42.9, 0) in the CA group vs 0.0 (−10.8, 0) in the LFM + AAD group at 9 months (*P* = 0.064), and 0.0 (−36.6, 0.0) in the CA group vs 0.0 (−11.9, 0.0) in the LFM + AAD group at 12 months (*P* = 0.017), again indicating a greater reduction in arrhythmia burden among patients treated with CA.

### Clinical outcomes

**Figure 4:**
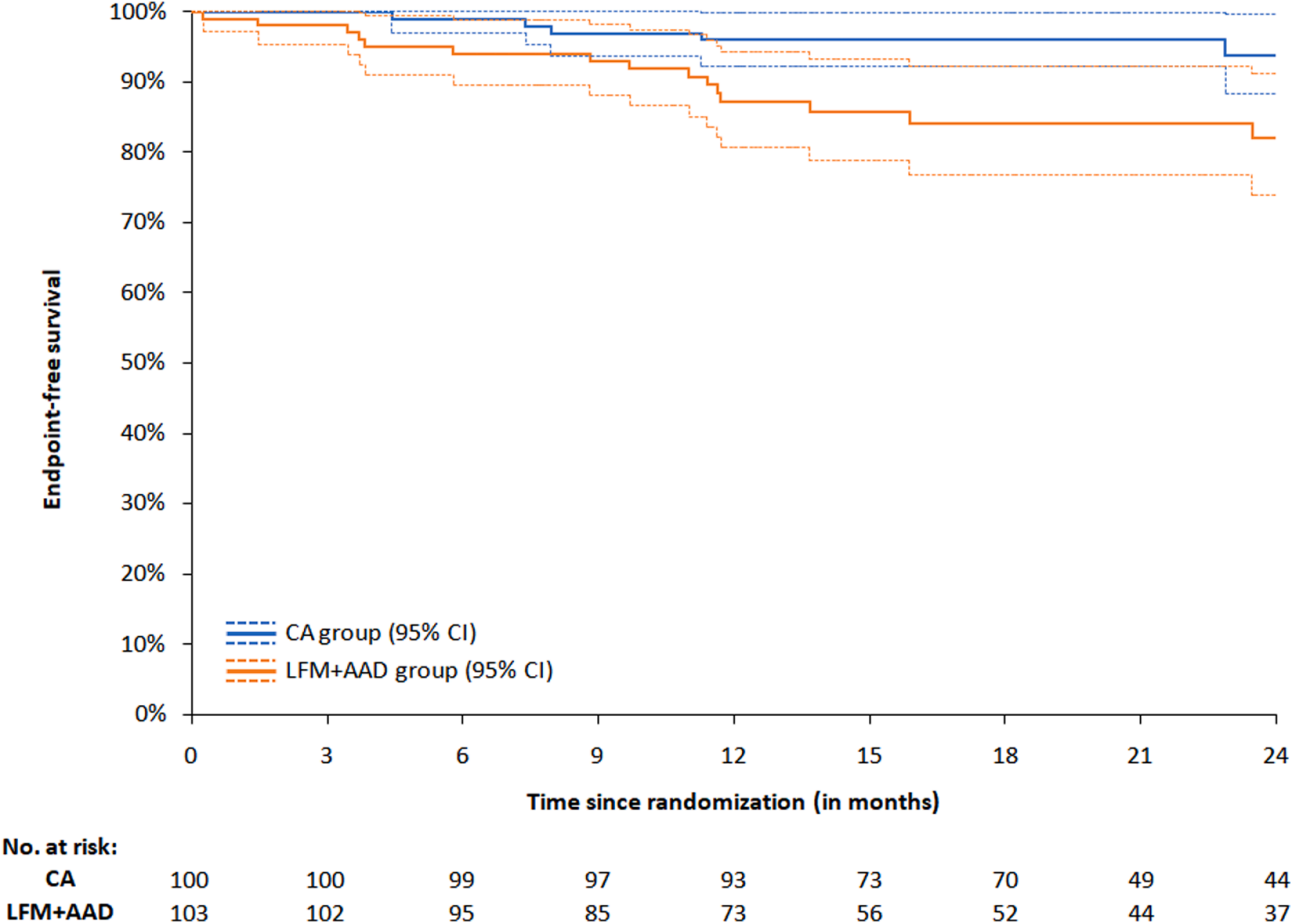
**AF-Related Endpoint-Free Survival During Follow-Up** Kaplan-Meier estimates of endpoint-free survival (freedom from AF-related hospitalization or emergency visit) from time of randomization are shown for the CA and LFM + AAD groups. Numbers at risk are displayed below the survival curves. Abbreviations as in Figure 1.

Hospitalization for AF recurrence and/or emergency department visit due to AF during the entire follow-up occurred in 7 (7%) patients in the CA group compared with 16 (15.5%) patients in the LFM + AAD group (Figure 4), resulting in a HR of 2.76 (95% CI: 1.13-6.77; *P* = 0.026). A composite endpoint of stroke, CV death, or HF hospitalization occurred in 1 (1.0%) patient in the CA group and 2 (1.9%) patients in the LFM + AAD group (*P* = 0.99).

Non–AF-related hospitalizations were not included in the predefined clinical endpoints. A brief summary is provided in the Supplemental Table 4.

## Discussion

Both treatment strategies, CA and lifestyle management combined with AADs, were associated with a decrease in AF burden over follow-up; however, the magnitude of reduction was substantially greater with CA. Furthermore, AF-related hospitalizations or emergency visits were lower in the CA group compared with LFM combined with AADs.

A key novelty of the present subanalysis is the evaluation of AF burden in the per-protocol population, which allows a clearer interpretation of rhythm outcomes compared with the original ITT analysis.

The original PRAGUE–25 analysis was according to the ITT principle. Although CA was superior in terms of AF recurrence in the time-to-first event analysis, AF burden at the 12-month follow-up did not differ between the groups. However, 15 patients underwent crossover during the first year. Crossovers did not affect the results of the primary time-to-event analysis, as crossovers were triggered by AF recurrences and patients were censored at the time of the first recurrence event.

In contrast, crossovers (with 1 exception, all from the LFM + AAD group into the CA group) significantly affected the results of AF burden assessed by Holter monitoring during follow-up, as these crossover patients were not excluded from later AF burden analysis. Therefore, the ITT analysis artificially favored patients in the LFM + AAD group, because AF burden at each analyzed milestone was affected not only by the effect of the LFM + AAD strategy, but also by the CA.

Therefore, the present per-protocol analysis includes only AF burden data from patients who remained in the LFM + AAD or CA group, and as such, it reflects the true effect of the particular treatment strategy. Although AF burden decreased at each milestone compared with baseline in both groups, the decrease of AF burden in the CA group was substantially greater than in the LFM + AAD group at each assessed milestone, whether evaluated as absolute values or as the difference between AF burden at a given milestone and baseline.

As demonstrated in recent studies on AF, AF burden represents a clinically more meaningful parameter than simple AF recurrences. AF burden has been consistently recognized as the most important aspect related to AF-related clinical outcomes. In the CIRCA–DOSE study, patients with an AF burden of ≤0.1% had rates of health care utilization comparable with patients completely free of recurrence. Disease-specific quality of life was significantly impaired with AF episode durations >24 h or AF burdens >0.1% compared with patients free of recurrence.^6^ In addition, higher AF burdens have been associated with an increased risk of stroke.^7^

Although studies comparing anticoagulation with aspirin have shown a broadly similar relative reduction in stroke risk in patients with clinical and device-detected subclinical AF, the absolute annual risk of stroke has been consistently lower in patients with subclinical AF, characterized by low AF burden, compared to patients with clinically manifest AF.^8^^,^^9^ Beyond AF-related events, AF burden also appears to be relevant for HF or AF-related outcomes.

Evidence linking AF burden to the development of HF was provided by a large cohort of nearly 40,000 patients with cardiac implantable electronic devices. Among individuals without HF at baseline, higher AF burden was independently associated with incident HF.^10^ The association showed a clear dose-response relationship and was consistent across sensitivity analyses, supporting the use of AF burden as a clinically relevant measure of arrhythmic burden rather than a dichotomous phenomenon.

Unfortunately, data evaluating the effect of weight loss or comprehensive LFM on AF burden remain limited. AF burden was not reported in the LEGACY or ARREST–AF studies.^2^^,^^3^ Importantly, AF burden was the primary endpoint in the SORT–AF trial assessing the effect of LFM after CA for AF. In this study, patients with AF and BMI between 30 to 40 kg/m^2^ underwent CA and were subsequently randomized to a targeted weight reduction program or standard care. CA was associated with a substantial reduction in AF burden in both groups (from 21.6% ± 36.0% to 3.7% ± 12.5% in the weight reduction group, and from 22.4% ± 36.8% to 4.2% ± 11.3% in controls), whereas further targeted weight reduction did not result in any additional significant reduction compared with controls.

The between-group difference in AF burden between 3 and 12 months, expressed as the difference in means (95% CI), was 0.00507% (−0.0373% to 0.0475%).^11^ These data suggest that the magnitude of AF-burden reduction achieved with CA markedly exceeded the effect of weight loss alone. Consequently, in the SORT–AF cohort of 67 patients, the reduction in AF burden achieved by CA may have already been so substantial that any smaller incremental effect of targeted weight loss could not be statistically demonstrated.

Consistent with these findings, clinical outcome–based data on weight reduction should be interpreted with caution. Further illustrating this, a recent study by Vermeer et al^12^ reported a lower incidence of clinical rhythm-control interventions, such as cardioversions and repeat ablations, in patients achieving weight reduction. However, neither AF burden nor AF recurrences were reported. The rhythm-related outcomes that were included, namely referral for electrical cardioversion or redo ablation, were limited to investigator-driven clinical decisions in an unblinded setting. Therefore, although these data support a potential association between weight loss and clinical outcomes, they do not allow any conclusions regarding the quantitative effect of weight reduction on AF burden.

The prognostic relevance of AF burden has also been demonstrated for major CV outcomes, including stroke, CV death, and HF hospitalizations. In the EAST–AFNET 4 trial, AF burden below the median was associated with significantly lower rates of CV death, stroke, or unplanned hospitalization for HF or acute coronary syndrome compared to patients with high AF burden. Based on intermittent electrocardiogram recordings, the incidence of CV death, stroke, or hospitalization for worsening HF rose from approximately 2.0 to 4.5 events per 100 person-years across AF-burden quartiles, and each 1% increase in AF burden corresponded to a 4% to 5% increase in the risk of CV death, stroke, or HF hospitalization.^13^

In our study, the incidence of MACEs did not differ between groups. However, the study was not adequately powered to compare these endpoints; a substantially larger cohort and longer follow-up would have been needed. In contrast, the rate of AF-related hospitalization or emergency visits was lower in CA patients compared to LFM + AAD patients. Although AF-related hospitalizations or emergency visits are less clinically significant than traditional MACE, they are substantially more common and can worsen patients’ quality of life and increase the economic burden of the health care system.

Several reports have demonstrated associations between AF burden, CA, AF-related hospitalizations, or unplanned hospital visits.^14^^,^^15^ For example, Norton et al^16^ showed that the incidence of emergency department visits and AF-related hospitalizations was higher among obese AF patients than nonobese AF patients. In the CENT study conducted by an Australian group of investigators, a risk factor modification (RFM) program in obese AF patients was associated with fewer unplanned AF-related visits, hospitalizations, or cardioversions.

However, the CENT study did not include CA patients as the control group; therefore, a direct comparison of the effect of RFM vs CA on AF-related outcomes could not be made. Even more importantly, the study may have been substantially biased by the composition of its control group.^17^ A dedicated RFM program was offered to all eligible obese AF patients; those who accepted the program formed the intervention group, whereas those who declined served as controls. This nonrandomized control group, consisting of patients who were inherently less motivated, represents the main limitation of the study. Patient motivation is a fundamental prerequisite for treatment success. Motivated patients may differ from less motivated individuals in several important aspects, including treatment adherence, psychological profile, non-CV comorbidities, and socioeconomic status.

Several reports have documented reductions in AF-related hospitalizations, cardioversions, or emergency department visits after CA. In early persistent AF, lower AF burden 1 year postablation was associated with greater improvement in AF-related symptoms and reduced AF-related health care utilization.^14^^,^^15^ CA has also been associated with a significant decrease in emergency visits or hospitalizations for AF compared with the period before the procedure.^18^^,^^19^ Similarly, in the EARLY–AF trial, CA compared to drug therapy translated into a reduction in clinically relevant AF-related health care utilization.^20^ Consistent with these findings, our patients who underwent CA had reduced AF burden as well as fewer AF-related hospitalizations or emergency visits.

### Study limitations

The original PRAGUE–25 trial was powered to compare CA with an LFM + AAD strategy, but was not powered for subgroup per-protocol analyses. Therefore, all results presented here should be interpreted as post hoc findings without a dedicated power calculation. Not all risk factors (eg, obstructive sleep apnea) were addressed in the LFM + AAD group. Our study focused on the 12-month follow-up postblanking period, and the effects of weight reduction would likely become more apparent over longer follow-ups. In addition, only a minority of patients in the LFM + AAD group received glucagon-like peptide-1 receptor agonists, which limited our ability to evaluate their specific contribution to outcomes. Finally, the occurrence of cardioversions before scheduled Holter monitoring, which may have influenced Holter results, was not compared between the groups.

Perspectives COMPETENCY IN MEDICAL KNOWLEDGE**:** In obese patients with AF, CA was associated with a greater reduction in AF burden compared with a treatment strategy based on LFM combined with AAD therapy. These findings support consideration of CA as an effective rhythm control strategy even in patients with obesity (BMI 30-40 kg/m^2^), a population in which conservative approaches are frequently preferred in routine clinical practice.

**TRANSLATIONAL OUTLOOK:** Future studies should evaluate whether pharmacologic weight-loss therapies, particularly glucagon-like peptide-1 receptor agonists, may modify AF burden and improve clinical outcomes in obese patients with AF, either as standalone strategies or in combination with CA.

## Conclusions

In obese patients with AF, AF burden decreased to a greater extent after CA than with treatment based on LFM plus AADs. As a result, CA was also associated with a reduced need for AF-related hospitalizations or emergency visits.

## Funding support and author disclosures

The study was supported by the research grant UNCE/24/MED/015 provided by Charles University, by the research grant Nr. NU21-02-00388 provided by the Ministry of Health of the Czechia, and by the Charles University Research program “Cooperatio-Cardiovascular science”. The authors have reported that they have no relationship relavant to the contents of this paper to disclose.

## References

[1] Wong C.X., Sullivan T., Sun M.T. (2015). Obesity and the risk of incident, post-operative, and post-ablation atrial fibrillation: a meta-analysis of 626,603 individuals in 51 studies. JACC Clin Electrophysiol.

[2] Pathak R.K., Middeldorp M.E., Meredith M. (2015). Long-term effect of goal-directed weight management in an atrial fibrillation cohort: a long-term follow-up study (LEGACY). J Am Coll Cardiol.

[3] Pathak R.K., Middeldorp M.E., Lau D.H. (2014). Aggressive risk factor reduction study for atrial fibrillation and implications for the outcome of ablation: the ARREST-AF cohort study. J Am Coll Cardiol.

[4] Osmancik P., Roubicek T., Havranek S. (2025). Catheter ablation vs lifestyle modification with antiarrhythmic drugs to treat atrial fibrillation: PRAGUE-25 trial. J Am Coll Cardiol.

[5] Osmancik P., Havránek S., Bulková V. (2022). Catheter ablation versus antiarrhythmic drugs with risk factor modification for treatment of atrial fibrillation: a protocol of a randomised controlled trial (PRAGUE-25 trial). BMJ Open.

[6] Andrade J.G., Deyell M.W., Macle L. (2023). Healthcare utilization and quality of life for atrial fibrillation burden: the CIRCA-DOSE study. Eur Heart J.

[7] Al-Khatib S.M., Thomas L., Wallentin L. (2013). Outcomes of apixaban vs warfarin by type and duration of atrial fibrillation: results from the ARISTOTLE trial. Eur Heart J.

[8] Healey J.S., Lopes R.D., Granger C.B. (2024). Apixaban for stroke prevention in subclinical atrial fibrillation. N Engl J Med.

[9] Kirchhof P., Toennis T., Goette A. (2023). Anticoagulation with edoxaban in patients with atrial high-rate episodes. N Engl J Med.

[10] Steinberg B.A., Li Z., O'Brien E.C. (2021). Atrial fibrillation burden and heart failure: data from 39,710 individuals with cardiac implanted electronic devices. Heart Rhythm.

[11] Gessler N., Willems S., Steven D. (2021). Supervised obesity reduction trial for AF ablation patients: results from the SORT-AF trial. Europace.

[12] Vermeer J., Vinck-de Greef T., van den Broek M. (2026). Improving outcomes of atrial fibrillation ablation by integrated personalized lifestyle interventions: a randomized controlled trial. Eur Heart J.

[13] Zeemering S., Borof K., Schotten U. (2025). Estimated atrial fibrillation burden on early rhythm-control and cardiovascular events in the EAST-AFNET 4 trial. EClinicalMedicine.

[14] Yeo M., Lee S.R., Choi J. (2025). Atrial fibrillation burden and symptom, quality of life, and healthcare resource utilization after cryoballoon ablation in persistent atrial fibrillation. Europace.

[15] Lee S.R., Choi E.K., Lee K.Y. (2025). Reduction of AF burden after cryoballoon ablation in patients with early persistent AF: the COOL-PER trial. JACC Clin Electrophysiol.

[16] Norton J., Foy A., Ba D.M. (2024). Obese patients with new onset atrial fibrillation/flutter have higher risk of hospitalization, cardioversions, and ablations. Am Heart J Plus.

[17] Pathak R.K., Evans M., Middeldorp M.E. (2017). Cost-effectiveness and clinical effectiveness of the risk factor management clinic in atrial fibrillation: the CENT study. JACC Clin Electrophysiol.

[18] Song Q., Tan H., Yang B. (2024). Quality of life and safety outcomes after first-line treatment of symptomatic AF with cryoablation or drug therapy: a meta-analysis of randomized controlled trials. Rev Cardiovasc Med.

[19] Dhande M., Barakat A., Canterbury A. (2023). Cardiovascular hospitalizations and resource use following atrial fibrillation ablation. J Am Heart Assoc.

[20] Andrade J.G., Deyell M.W., Macle L. (2023). Progression of atrial fibrillation after cryoablation or drug therapy. N Engl J Med.

