# Supplementary material for: Atrial Fibrillation Burden and Clinical Outcomes After Catheter Ablation vs Lifestyle Modification With Antiarrhythmic Drugs
Source: JACC Adv. 2026 Aug 6;5(9):103109. doi: 10.1016/j.jacadv.2026.103109 (PMC13475505; doi:10.1016/j.jacadv.2026.103109)
Supplement: Supplementary file 1 [file mmc1.docx]

**Supplemental Appendix**

*Atrial Fibrillation Burden and Clinical Outcomes after Catheter Ablation versus Lifestyle Modification with Antiarrhythmic Drugs*

**Contents**

Page 2:	Supplemental Table S1. Adjusted sensitivity analysis of AF burden

Page 3:	Supplemental Table S2. Transitions in Holter-derived AF pattern from baseline to 12 months

Page 4:	Supplemental Table S3. Comparison of VO₂ max and quality-of-life outcomes between groups at 12 months, adjusted for baseline values, in the per-protocol populations

Page 5:	Supplemental Table S4. Non–AF-related hospitalizations and treatment-related adverse events

**Supplemental Table S1. Adjusted sensitivity analysis of AF burden**

| **Time point** | **Adjusted treatment effect (β, 95% CI)** | **p-value** | **n (CA)** | **n (LFM+AAD)** |
| --- | --- | --- | --- | --- |
| 6 months | −0.62 (−0.98 to −0.25) | <0.001 | 95 | 96 |
| 9 months | −0.42 (−0.79 to −0.05) | 0.026 | 97 | 86 |
| 12 months | −0.42 (−0.79 to −0.04) | 0.029 | 96 | 83 |

Standard beta regression was used as an adjusted sensitivity analysis of AF burden, with treatment group as the main predictor and baseline AF burden and baseline AF type (paroxysmal vs non-paroxysmal) as covariates. AF burden was rescaled to proportions, and boundary values of 0 and 1 were replaced by 0.001 and 0.999, respectively, before model fitting. Negative values indicate that the AF burden at the corresponding milestones was lower in the CA group compared with the LFM+AAD group.

**Supplemental Table S2**

**Transitions in Holter-derived AF pattern from baseline to 12 months**

| **Transition** | **CA** | **LFM+AAD** |
| --- | --- | --- |
| AF burden 100% → AF burden 100% | 4 (4.2%) | 11 (13.3%) |
| AF burden 100% → AF burden <100% | 19 (19.8%) | 10 (12.0%) |
| AF burden <100% → AF burden 100% | 4 (4.2%) | 1 (1.2%) |
| AF burden <100% → AF burden <100% | 69 (71.9%) | 61 (73.5%) |

An AF burden of 100% indicates continuous AF throughout the 7-day Holter recording; an AF burden <100% indicates non-continuous AF. Results are presented as counts (percentages) within each treatment group.

The distribution of transitions did not indicate an increased progression to continuous AF in the LFM+AAD group.

**Supplemental Table S3.**

**Comparison of VO₂ max and quality-of-life outcomes between groups at 12 months, adjusted for baseline values, in the per-protocol populations**

| **Outcome** | **Adjusted difference, CA − LFM+AAD (β, 95% CI)** | **p-value** | **n (CA)** | **n (LFM+AAD)** |
| --- | --- | --- | --- | --- |
| VO₂ max | −1.54 (−2.72 to −0.36) | 0.011 | 86 | 73 |
| AFEQT total score | 3.67 (−0.27 to 7.61) | 0.068 | 96 | 83 |

Analyses were performed in a *per-protocol* population defined as patients without cross-over and with available baseline and 12-month measurements for the respective outcome. Adjusted analyses were conducted using linear regression models (ANCOVA approach), with the 12-month value as the dependent variable and baseline value as a covariate. β represents the adjusted difference calculated as CA − LFM+AAD; negative values indicate lower adjusted 12-month values in the CA group, whereas positive values indicate higher adjusted 12-month values in the CA group.

As shown by the results, VO₂ max values were significantly higher in the LFM+AAD group compared with the CA group, even after adjustment for baseline VO₂ max values. In contrast, the difference in AFEQT between the groups was not significant.

AFEQT = Atrial Fibrillation Effect on Quality-of-Life Questionnaire.

**Supplemental Table S4**

**Non–AF-related hospitalizations and treatment-related adverse events**

| **Event** | **CA (n)** | **LFM+AAD (n)** |
| --- | --- | --- |
| Hospitalization due to treatment-related complication (bradyarrhythmia requiring pacemaker) | 0 | 1 |
| Major procedure-related complication (TIA*) | 1 | 0 |
| Minor procedure-related complication (pseudoaneurysm) | 1 | 0 |
| Non-hospitalized adverse events (syncope/presyncope) | 0 | 3 |
| Sudden unexplained death (out-of-hospital) | 0 | 1 |

* Transient ischemic attack (TIA) occurred during the index hospitalization and was not counted as a separate hospitalization.
